# A Clinical Memoir on Strangulated Hernia

**Published:** 1860-05

**Authors:** 


ART. XI.—*A Clinical Memoir on Strangulated Hernia, with the Author's Practice: to which are added Remarks on Obstruction of the Bowels from other Causes; and a Postscript.* By GEORGE MACILWAIN. 8vo., pp. 120. London, 1858.

"NON semper ea sunt quæ videntur; dĕcĭpit frons prima multos." The present quotation is admirably illustrative of our opinion of Mr. Macilwain's work; when we ordered it through our foreign bookseller, we supposed, from the interesting nature of its title, that we should soon be put in possession of a great treasure, especially when we looked at that part of it marked, "with the author's practice." We had formed a sort of personal acquaintance, and one by no means of a disagreeable kind, with the author from a perusal of his treatise on "Medicine and Surgery one Inductive Science," and several minor efforts of his pen; but, truly "things are not always what they seem to be—first appearances deceive many," and we plead guilty to the soft impeachment of having been prodigiously humbugged by our purchase. The volume is about as jejune a performance as could well be conceived of; and when we inform the reader that the "author's practice" consists in the proscription of anæsthetics in the reduction of strangulated hernia, and in the employment of tobacco injections, we think he will agree with us that it would be a great blessing if another Paracelsus could arise to commit it to the flame. We pity, from the bottom of our heart, a man who can eschew so beneficent an agent as ether or chloroform under such circumstances, and deliberately set about the employment of so dangerous a remedy as tobacco. Mr. Macilwain is evidently surcharged with the most unpardonable prejudice in regard to the use of anæsthetics in the treatment of strangulated hernia. It is quite amusing to hear him talk on this subject. "Of chloroform, in strangulated hernia, I have no experience;" and, considering that his Memoir was published in the year of grace 1858, it is not at all likely that he ever will have any. Should he live twenty-five years longer, no treatment, however skillfully directed or perseveringly continued, will remove the thick scum which now covers his eyes in regard to this class of agents. Mr. Macilwain is evidently a conservative surgeon. When he entered the profession, thirty-five or forty years ago, he learned that tobacco was occasionally useful in promoting the reduction of strangulated hernia; and, true to the teachings of that period, he still persists in the villainous treatment, a treatment no longer employed by any Christian practitioner.

The following are our author's *reasons* for his rejection of chloroform in the affection under consideration: "With regard to chloroform, we know that it has produced lamentable results, altogether independently of those cases in which it has proved fatal. Two or three years ago, in a case of lithotomy, performed without chloroform, I went into the next room the moment I had removed the stone, to inform the mother that the operation was over, when the first thing she said was, 'Oh, sir, I am so glad you did not use chloroform!' And upon asking why, she gave me a very lamentable history of a daughter who was then laboring under mental aberration, which, she said, was attributed by the physicians to the use of chloroform." Pray, Mr. Macilwain, did you never know of patients having been killed by tobacco injections in attempts at reducing strangulated hernia, or by opium administered for the relief of pain, or by tartar emetic for the purpose of vomiting a man affected with disorder of the stomach and liver? Or, if afraid of chloroform, why could you not substitute ether? Surely no old, ignorant woman could object to that on account of its supposed, and merely supposed, injurious impression on the brain?

It would be somewhat difficult, without a more profound knowledge than is conveyed by his lessons, to determine the peculiarity of the "author's practice" in strangulated hernia. The following passage will, perhaps, afford some idea of its character:—

"When called to a case of hernia, I endeavor to determine the probability of its proving reducible. If I determine in the affirmative, I apply the taxis, with the cautions I have hinted, at once. If I think it will prove irreducible, then, subject to the conditions which I have mentioned, I administer the tobacco. The surgeon should always superintend the administration of the tobacco himself; and immediately that the required lassitude is produced (if the hernia do not recede of itself, which sometimes happens,) he should endeavor to reduce it. If many persons are in the room, I request them to retire; they only disturb the patient. For the same reason, pupils in a hospital should not be allowed to crowd round a patient's bed. There is nothing to be learned by it, but much that may foil you by raising the alarm and excitement which all unnecessary circumstance occasions. If the tobacco do not succeed, the patient should be allowed to recover from its effects, and then the hernia liberated without delay. The removal of the patient to an operating theatre, or another apartment, is objectionable, except for purposes of improved light or something strictly relevant to the requisitions of the operator. In fact, all unnecessary motion should be avoided."

We are glad to find, after so much that is objectionable in Mr. Macilwain's practice, that he is a warm advocate for repose of the bowels for several days after the operation for strangulated hernia. The practice of Travers, Lawrence, and others, of administering purgatives soon after the restoration of the parts cannot be too severely or pointedly reprehended. Rest of the intestinal tube for many hours is indispensable to the prevention of peritonitis. It is a practice which we have zealously advocated in our teachings for the last twenty years.

We candidly confess we do not see why Mr. Macilwain should have inflicted this Memoir upon the profession. It is greatly to be regretted that so much fine paper and good ink should have been wasted upon such trash as is contained in its pages, without a solitary redeeming quality as to its intrinsic merit, or even the value of "the author's practice."

